# Editorial: Challenges, opportunities, and actions for improved maternal and child nutrition

**DOI:** 10.3389/fnut.2025.1580113

**Published:** 2025-03-20

**Authors:** Monica Ancira-Moreno, Sonia Hernández-Cordero

**Affiliations:** ^1^Health Department, Universidad Iberoamericana, Mexico City, Mexico; ^2^Observatorio Materno Infantil (OMI), Universidad Iberoamericana, Mexico City, Mexico; ^3^Research Center for Equitable Development EQUIDE, Universidad Iberoamericana, Mexico City, Mexico

**Keywords:** maternal nutrition, child nutrition, pregnancy, childhood, malnutrition, interventions

Maternal and child nutrition remains a significant public health challenge globally, especially in low- and middle-income countries. Despite advancements in understanding the importance of nutrition during the first 1,000 days, implementation of interventions and their outcomes show considerable gaps. This Research Topic features nine original research articles that address critical issues, including a description of the magnitude of the problem of malnutrition in countries, the inadequate consumption of vitamins and minerals during pregnancy, the response to prevent malnutrition in emergency settings, and the high consumption of ultra-processed foods during the first 1,000 days and its impact on health and cognitive development and the effects of integrated health interventions on malnutrition. The studies urgently call for targeted nutritional interventions, comprehensive policy reforms, and community-driven strategies to combat malnutrition and its consequences. They align with target 2.2: “End all forms of malnutrition” (1) of the United Nations' Sustainable Development Goals for 2030.

The Research Topic aims are to (i) synthesize globally recognized definitions and tools for assessing nutritional quality and health outcomes; (ii) explore epidemiological trends related to malnutrition and the effects of ultra-processed foods on cognitive development; (iii) evaluate the effectiveness of nutrition interventions in various contexts; and (iv) highlight the socio-cultural, economic, and environmental determinants influencing caregivers' health-seeking behaviors and pregnancy outcomes.

The importance of promoting and ensuring the adequate intake of vitamins and minerals in the diet of toddlers (1–2 years) and preschoolers (3–5 years) is highlighted by the study conducted by Burgard et al. in Germany. In this study, in addition to showing inadequate vitamin D, iodine, and iron intake, there are also high intakes of saturated fatty acids. The authors analyzed data from the representative cross-sectional Children's Nutrition Survey KiESEL conducted between 2014 and 2017. The authors underscore the need for further research into extending vitamin D supplementation beyond infancy and call for enhanced public health initiatives to increase the use of iodized salt and reduce the intake of SFA and sugars in children's diets to address these imbalances.

The findings of Zhang et al.'s study underline the critical global challenge in child nutrition that transcends geographical boundaries. This study examined child malnutrition and its associated factors among 18,503 children aged 3 to 14 in Beijing and Tangshan from September 2020 to January 2022. The prevalence of malnutrition was 10.93%. Multivariable analysis identified seven significant predictors: parental education (OR 1.52), family income (OR 1.23), fast-food intake frequency (OR 1.14), night meals frequency (OR 1.09), eating speed (OR 1.01), and maternal and paternal obesity (both OR 0.97). These factors strongly predicted child malnutrition, emphasizing the need to address these risk factors to improve child nutrition and public health.

The study carried out by Naila et al. investigates the healthcare-seeking behaviors of caregivers for critically malnourished children under 5 years old among forcibly displaced Myanmar Nationals (FDMNs) in Bangladesh. The study's main conclusion stresses the importance of culturally sensitive interventions to promote caregiver awareness of severe wasting, enhanced healthcare accessibility, and increased community volunteer engagement, which have the potential to facilitate early identification of severely wasted children and mitigate delays in treatment. These insights align with findings from studies in Germany and China, emphasizing the need to address socio-cultural factors to enhance maternal and child nutrition in vulnerable populations.

Furthermore, Liu Y. et al. examined the associations between nutritional trace metals (NTMs) during pregnancy and the risk of preterm birth (PTB) in women experiencing recurrent pregnancy loss. The research demonstrated that higher maternal exposure to copper (Cu) and zinc (Zn) was inversely associated with PTB risk, suggesting that increased levels of these metals may lower the likelihood of PTB, with Cu identified as a significant factor. The study highlights the importance of NTMs in understanding PTB risk and offers insights for personalized care and preventive strategies to improve maternal and infant health outcomes.

This Research Topic also includes studies highlighting the effectiveness of health programs in various contexts, particularly in the most vulnerable settings. One such study by Pedrero-Tomé et al. evaluated a health program in the Tama health area of Bouza, Tahoua, and Niger, designed to combat high malnutrition rates in children under two amid severe food insecurity and high infant mortality. The program, involving 6,962 participants, included vaccinations, malaria prevention, and nutritional supplementation for children over 6 months. Results showed a decline in the proportion of children without anthropometric failure, dropping from 59.5% to 40.2% (*p* < 0.001). The study highlights the need for integrated approaches addressing both infectious diseases and malnutrition in vulnerable young children.

Similarly, the study by Sánchez-Martínez et al. focuses on the treatment of moderate acute malnutrition (MAM) in emergency settings, particularly in the Diffa region of Niger. It evaluates a simplified treatment protocol administered by Community Health Workers (CHWs) compared to the standard protocol provided by nursing staff. In a non-randomized controlled trial, the intervention group (*n* = 483) exhibited a significantly higher recovery rate (99.6% vs. 79.56%, *p* < 0.001), faster recovery times, and better anthropometric gains compared to the control group (*n* = 181). Additionally, treatment coverage in the intervention group increased from 28.8% to 84.9%, declining in the control group from 25.3% to 13.6%. Importantly, recovery rates were similar for children treated by CHWs and nursing staff, indicating that the simplified protocol can be effectively administered in emergencies. The findings highlight the need for innovative actions for the prevention and treatment of MAM in emergency contexts, integrating the participation of CHWs with the use of valid and precise indicators, such as Mid-upper arm circumference, as a risk and monitoring indicator of the nutritional status of children.

This topic also highlights the pervasive influence of ultra-processed food consumption across different life stages, emphasizing the need for targeted interventions to improve dietary habits and health outcomes for mothers and children. Granich-Armenta et al. investigated the contribution of ultra-processed foods (UPFs) to total energy intake during pregnancy, focusing on pre-gestational BMI and hemoglobin (Hb) levels in the MAS-Lactancia Cohort in Mexico. UPFs comprised about 27% of total energy intake during the second and third trimesters, with no significant changes between these periods. Women with pre-gestational obesity and low Hb levels had higher UPF energy contributions (23.1% to 44.7%) compared to those with normal BMI and higher Hb (18% to 38.6%). The high intake of sugars, saturated fats, and sodium raises maternal and child health concerns, highlighting the need for better nutrition during pregnancy.

Along the same lines, Liu S. et al. investigated the harmful effect of consuming ultra-processed foods, which goes beyond health issues, showing how these products have a negative impact on cognitive function in preschool children in China. The study examined 325 children aged 4–7 from the Guangxi Zhuang Birth Cohort. It used interviews and a Food Frequency Questionnaire to evaluate their intake of ultra-processed foods like candy and sugary drinks. The findings showed that frequent consumption of candy and sweet baked goods was significantly linked to lower full-scale IQ scores, with candy also increasing the risk of cognitive deficits. Additionally, children who consumed more than two types of ultra-processed foods had lower Verbal Comprehension Index scores.

All the studies above underscore the importance of comprehensive recommendation guidelines prioritizing maternal nutrition. The Ancira-Moreno et al. study contributed to emphasizing this dimension. This research sought to identify clinical practice guidelines (CPGs) addressing maternal malnutrition prevention, diagnosis, and treatment. Guidelines for women in the preconception period were reviewed, and their quality was assessed using the AGREE II tool. Of the 30 guidelines screened, 20 were fully evaluated, with an overall quality score of 73%. Only 55% were classified as high quality (score > 70%). The highest scores were in the “Scope and Purpose” (98.5%) and “Clarity of Presentation” (93%) domains. The study underscores the need to improve the quality of guidelines for managing women's malnutrition.

## Challenges, opportunities, and strategic actions

Advancing maternal and child nutrition requires addressing persistent and complex challenges, including socio-economic inequalities, gaps in healthcare infrastructure, and cultural determinants of health behaviors. These challenges are compounded by the pervasive influence of ultra-processed foods, insufficient adherence to clinical practice guidelines, and limited integration of evidence-based interventions into healthcare systems. However, these barriers also create opportunities for transformative solutions. Strategic actions include the development of robust policies that integrate nutrition-specific and nutrition-sensitive interventions, applying advanced technologies to enhance monitoring and evaluation systems, and strengthening multisectoral collaboration to optimize resource allocation and impact. Furthermore, prioritizing the training and deployment of community health workers, alongside scaling culturally sensitive approaches, can enhance early detection and treatment of malnutrition. By implementing these targeted and evidence-driven strategies, substantial progress can be achieved in mitigating the global burden of maternal and child malnutrition.
